# Gold Nanoparticle-Based Plasmonic Biosensors

**DOI:** 10.3390/bios13030411

**Published:** 2023-03-22

**Authors:** Enrico Ferrari

**Affiliations:** Department of Life Sciences, University of Lincoln, Lincoln LN6 7TS, UK; eferrari@lincoln.ac.uk

**Keywords:** molecular diagnostics, naked-eye detection, point-of-care testing

## Abstract

One of the emerging technologies in molecular diagnostics of the last two decades is the use of gold nanoparticles (AuNPs) for biosensors. AuNPs can be functionalized with various biomolecules, such as nucleic acids or antibodies, to recognize and bind to specific targets. AuNPs present unique optical properties, such as their distinctive plasmonic band, which confers a bright-red color to AuNP solutions, and their extremely high extinction coefficient, which makes AuNPs detectable by the naked eye even at low concentrations. Ingenious molecular mechanisms triggered by the presence of a target analyte can change the colloidal status of AuNPs from dispersed to aggregated, with a subsequent visible change in color of the solution due to the loss of the characteristic plasmonic band. This review describes how the optical properties of AuNPs have been exploited for the design of plasmonic biosensors that only require the simple mixing of reagents combined with a visual readout and focuses on the molecular mechanisms involved. This review illustrates selected examples of AuNP-based plasmonic biosensors and promising approaches for the point-of-care testing of various analytes, spanning from the viral RNA of SARS-CoV-2 to the molecules that give distinctive flavor and color to aged whisky.

## 1. Introduction

The use of gold nanoparticles (AuNPs) in plasmonic biosensors for molecular diagnostics has a relatively short history, dating back to the early 2000s. AuNP-based biosensors have been developed primarily for the detection of small molecules, DNA, and proteins using the surface plasmon resonance (SPR) properties of AuNPs for the highly sensitive and selective detection of target molecules [1,2,3,4]. Over the years, advances in nanotechnology and materials science led to the development of increasingly sophisticated biosensors based on AuNPs, for example, the integration of AuNP-based readouts within microfluidic systems [5,6] or the use of non-spherical nanoparticles with distinctive optical properties [7,8].

The COVID-19 pandemic has brought a renewed focus on using AuNP-based biosensors for molecular diagnostics. Due to their rapid and highly sensitive detection capabilities, AuNP-based biosensors have been used to develop point-of-care diagnostic tests for the SARS-CoV-2 virus that causes COVID-19 [9,10,11,12]. A variety of molecular diagnostic platforms, including lateral flow assays (LFAs) [13,14,15] and lab-on-a-chip devices [16,17], have been developed in the years affected by the COVID-19 pandemic and, especially the LFAs, have been instrumental in controlling the spread of the virus and have played a crucial role in global public health efforts.

**Figure 1:**
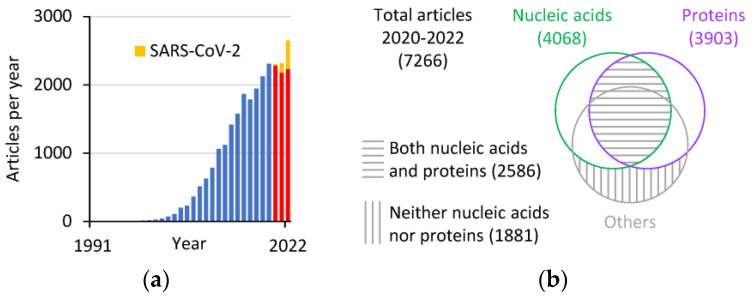
Bibliometric analysis of articles featuring AuNP-based biosensors (data from Scopus). (**a**) Articles per year that have “gold nanoparticle biosensor” or “detection” in the title, abstract, or keywords, excluding the words “lateral flow”, and limited to the “article” document type. The red bars refer to the years 2020–2022 characterized by the COVID-19 pandemic, with the yellow portion of the bars representing the articles mentioning “SARS-CoV-2” in the title, abstract, or keywords. (**b**) Venn diagram of the 2020–2022 articles from panel a grouped based on the presence or absence of the words DNA or RNA (nucleic acids) and protein in the title, abstract, or keywords.

A bibliometric analysis of data from the Scopus database (www.scopus.com, accessed on 9 February 2023) reveals that, since the first paper on the use of AuNPs for plasmonic biosensors was published in 1997 [18], the number of articles per year became two digits only two years later (Figure 1a). The number quickly reached three digits in 2004, four digits in 2011, and 2645 articles in 2022, the highest number of published articles per year on this topic so far. In the three years 2020–2022, which were characterized by the COVID-19 pandemic, the positive trend in publications appears to be unaffected, although an increasing proportion of the published articles focuses on the detection of SARS-CoV-2 molecules (22, 139, and 412 articles in the years 2020, 2021, and 2022, respectively). The analysis of the 7266 articles published in 2020–2022 reveals that the research is roughly equally split between the detection of nucleic acids (DNA and RNA) and proteins, with a substantial proportion of articles mentioning both (Figure 1b). A smaller but still sizable number of articles focuses on the detection of analytes other than nucleic acids or proteins.

The growing research trend suggests the continued development and use of AuNP-based biosensors, which will likely have a significant impact on the future of molecular diagnostics and will play a critical role in the fight against not only COVID-19 but also other diseases and infectious agents [19]. Building on the momentum that plasmonic biosensors are experiencing within human health, increased applications and the development of new dedicated biosensors are also likely to happen in animal and plant health [20,21], food safety [22], and environmental monitoring [23].

This review focuses on emerging molecular diagnostic methods for the rapid detection of nucleic acids and proteins, limited to where the assay readout mechanism is simply based on the optical properties of AuNPs in solution, thus excluding approaches covered elsewhere such as LFAs or lab-on-a-chip devices where the readout requires the immobilization of molecular components on functional surfaces or devices [24,25].

## 2. Optical Properties of Gold Nanoparticles

AuNPs exhibit unique optical properties that distinguish them from bulk gold or other materials. These optical properties are due to the large surface-to-volume ratio, high SPR, and interparticle interactions. The vivid colors of AuNP colloids make them a popular object of study for chemists and physicists [26] and a standard tool in many areas of biology and medicine [27]. SPR is a resonant interaction between light and metal nanoparticles that leads to strong absorption and scattering of light. SPR is influenced by the size, shape, and environment of the metal nanoparticles. The SPR of gold nanoparticles is tunable over a wide range of wavelengths, from the visible to near-infrared regions [28,29], although most plasmonic biosensors use spherical nanoparticles of tens of nanometers in size, which give typical SPR peaks around 515–560 nm. Besides molecular diagnostics, due to its sensitivity to the environment in which the AuNPs are suspended, SPR can be used to probe the refractive index of the surrounding medium, including studying the kinetics of molecules that transiently bind to AuNPs [30]. The calculation of the scattering properties of AuNPs of various sizes and shapes can be achieved computationally using the Mie scattering theory [31]. On the other hand, the distinctive scattering properties of AuNPs also allow for the determination of physical properties such as the particle size simply based on extinction spectra of the colloidal solution [32,33].

**Figure 2:**
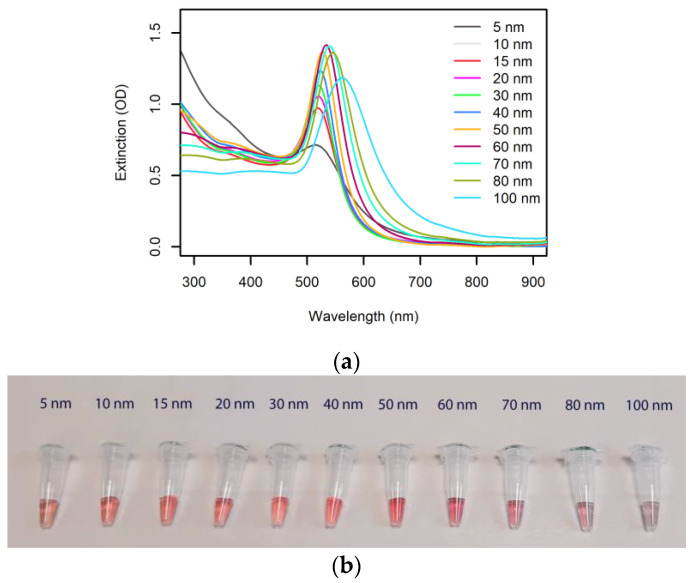
Optical properties of spherical AuNPs. (**a**) Extinction spectra (1 cm pathlength) of commercial 50 μg/mL citrate-capped AuNP colloids (nanoComposix, San Diego, CA, USA). (**b**) Photograph of the AuNP solutions from panel a.

The well-defined extinction peak wavelength and the high extinction coefficient of AuNPs are the basis of the use of AuNPs in plasmonic biosensors. Figure 2 shows the extinction spectra and representative photos of citrate-capped AuNPs in a range of sizes and at a gold concentration of 50 μg/mL.

The extinction peak wavelengths, specific to each AuNP size, are reported in Table 1, along with the relevant extinction coefficients. The very high extinction coefficients and the low concentrations required to produce a bright color well visible by the naked eye (1 OD in a 1 cm cuvette) highlight the unique potential that AuNPs have in deployable molecular diagnostics, where easy readout has to be combined with low limits of detection.

As a useful comparison, the food additive betanin, also known as beetroot red or E162, which is used as an intense red-violet dye with an absorption peak at 538 nm, has an extinction coefficient of 6 × 10^4^ M^−1^ cm^−1^ [34] and would require a concentration of 17 μM to provide the same intensity as the AuNPs listed in Table 1 (1 OD at 1 cm pathlength).

**Figure 3:**
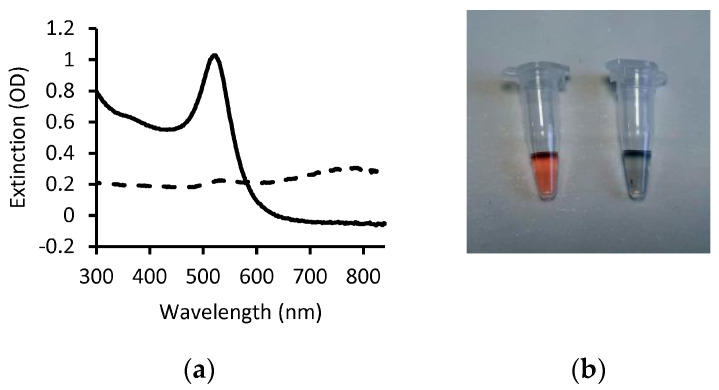
Change in AuNP optical properties due to aggregation. (**a**) Extinction spectra of dispersed 20 nm citrate-capped AuNPs (solid line) and of the same solution after adding NaCl (dashed line). The presence of charged ions destabilizes the colloid via screening of negative charges with consequent aggregation and loss of the SPR band. (**b**) Photograph of the AuNP solutions from panel a (dispersed, left; aggregated, right).

It is important to note that in absence of stabilization factors, such as repulsion due to net charge or steric hindrance due to polymers grafted on the nanoparticle surface, the van der Walls attractive forces between AuNPs tend to trigger aggregation, with subsequent loss of the SPR band and change in color of the colloidal solution [3]. AuNP aggregation, triggered by the binding of the analyte or as a consequence of a cascade of reactions that follow molecular recognition of the analyte, is the primary mechanism for obtaining a readout visible by the naked eye as the changes in the extinction spectrum and color of the solution are remarkable (Figure 3).

It is worth noting that, due to their well-defined plasmonic band, AuNPs are often used as a quencher (i.e., energy acceptor) in Förster resonance energy transfer (FRET). Unlike the extinction-based detection of AuNPs, FRET-based biosensors require instrumentation for readout and therefore are beyond the scope of this review and have been reviewed elsewhere [35].

Besides AuNPs, other metal nanoparticles such as silver, copper, aluminum, titanium, palladium, and platinum nanoparticles present plasmonic responses in the ultraviolet, visible, or infrared spectrum [36]. While these nanoparticles have interesting properties, they may not have the same level of stability, biocompatibility, or ease of synthesis and functionalization as AuNPs.

## 3. Synthesis and Bioconjugation of Gold Nanoparticles

Besides the interesting optical properties, AuNPs became one of the most popular nanomaterials for biosensors thanks to the availability of accessible, reliable, and affordable synthesis methods and facile surface chemistry. The latter is primarily enabled by the reactivity of thiol groups towards the gold surface or, alternatively, by the ability to passively adsorb biomolecules at the solid–liquid interface [26].

### 3.1. AuNP Synthesis

There are several methods for synthesizing AuNPs of different sizes and with different surface charges. The chemical reduction method is the most widely used. In this method, gold ions are reduced to nanoparticles by using reducing agents, such as sodium citrate, ascorbic acid, or hydrogen peroxide [37]. The size of the nanoparticles can be controlled by varying the concentration of the reducing agents. The surface charge of the nanoparticles can also be modified by adding stabilizing agents, such as surfactants or polymers [38]. Unlike the synthesis of other forms of plasmonic Au-based materials such as thin layers, the chemical reduction method for the synthesis of AuNPs does not require dedicated equipment, making it an accessible way to generate plasmonic structures.

Physical methods of synthesis involve the use of physical forces, such as thermal, mechanical, or laser irradiation, to produce AuNPs. For example, laser ablation is a physical method that involves the use of high-intensity laser pulses to vaporize bulk gold into nanoparticles [39].

In biological methods, bacteria, fungi, yeast, or plant extracts are used as bioreactors for the synthesis of AuNPs [40,41,42,43]. These microorganisms can reduce gold ions to nanoparticles using metabolic pathways, and the size and surface charge of the nanoparticles can be controlled by varying the culture conditions.

In template-assisted methods, gold ions are deposited onto a template material such as a porous material or a patterned polymer to form nanoparticles [44]. The size and shape of the nanoparticles can be controlled by the size and shape of the template pores or patterns.

In seed-mediated methods, pre-synthesized AuNP seeds are used to grow larger nanoparticles through the addition of gold ions [45]. The size of the nanoparticles can be controlled by varying the concentration of the gold ions and the reaction time.

### 3.2. AuNP Bioconjugation

The choice of method for AuNP bioconjugation depends on the desired specificity, stability, and functionality of the conjugates, as well as the properties of the biomolecules being attached. Decoration strategies vary enormously and have been reviewed elsewhere [46].

The simplest and most common method for attaching biomolecules to AuNPs is by direct adsorption. This method involves mixing AuNPs with a solution containing the biomolecules which adsorb onto the AuNP surface via physical (physisorption) or chemical (chemisorption) interactions.

Another common decoration method is via thiol-containing biomolecules which can form covalent bonds with AuNPs via their thiol groups [47]. This method is highly specific and efficient and results in stable conjugates. For example, antibodies can be attached to AuNPs through their Fc region, which contains a high density of thiol groups [48].

DNA can be attached to AuNPs through the formation of covalent bonds with functional groups, such as amine or thiol groups, on the DNA molecule or by hybridizing single-stranded DNA to complementary sequences attached to the AuNP surface [49].

Besides passive adsorption, proteins can be attached to AuNPs through a variety of chemical cross-linking methods [50], through the formation of covalent bonds with cysteine residues on the protein surface [51], or via genetically encoded tags that help to correctly orient the protein on the AuNP surface [52,53].

## 4. Molecular Mechanisms for the Detection of Biomolecules and Analytes Using Gold Nanoparticles as a Visual Readout

While the easy synthesis and the stability of gold colloids help to contain the costs and shelf-life of AuNP-based molecular diagnostic reagents, the versatile bioconjugation methods enable the binding of functional molecules which can interact specifically with the analytes. However, to trigger a change in the visual readout upon binding of the analyte, a molecular mechanism has to be in place, typically determining a change in color due to aggregation of the AuNPs in solution. This section describes the main molecular mechanisms that have been applied to trigger optical transitions of AuNP solutions to detect the presence of the target molecules, spanning from macromolecules, such as DNA, RNA, and proteins, to ions and small molecules. It is the combination of the high extinction coefficient of AuNPs and the ingenious strategies devised to convert molecular binding events into a visible readout that ultimately determines the remarkable sensitivity of AuNP-based plasmonic biosensors.

Here, selected examples from the literature are reported which either illustrate well the mechanism involved in the plasmonic diagnostic assays or represent recent promising advances in the detection of nucleic acids, proteins, and other analytes. The key biosensors described in the following sections are also summarized in Table 2.

### 4.1. Detection of Nucleic Acids

**Figure 4:**
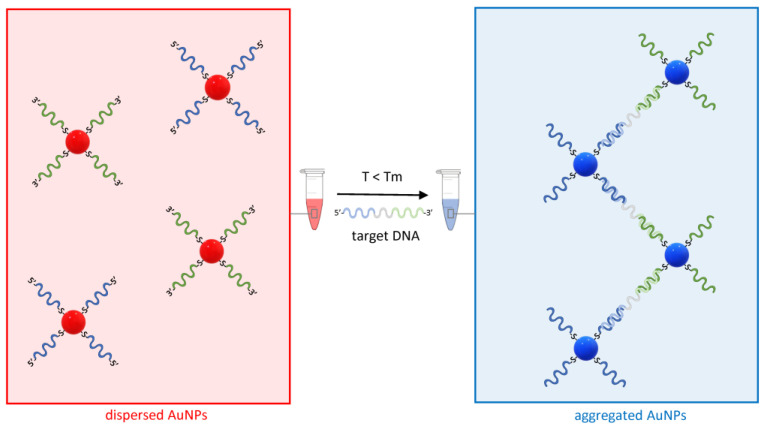
The principle of sandwich hybridization for the detection of nucleic acids using AuNP–ASOs. On the left, two distinct sets of AuNP–ASOs are represented by the blue and green ssDNA molecules conjugated via a thiol–gold bond to the AuNPs at the 5′ or 3′ end. In presence of a target DNA complementary to both ASOs and a temperature T below the melting temperature Tm of the ASOs, the AuNPs undergo aggregation, and the red color typical of a dispersed solution of AuNPs shifts towards the blue (right).

For their unique ability to specifically hybridize to a complementary strand, single-strand nucleic acid oligonucleotides were the first macromolecules used to assemble AuNPs into controlled complexes [49]. Shortly, the concept was exploited for the detection of specific DNA sequences [18], including the identification of a single base mismatch, deletion, or insertion [60]. AuNP–DNA oligonucleotide conjugates have been applied to the detection of a range of nucleic acid targets using different hybridization and readout strategies which have been extensively reviewed elsewhere [61]. As a paradigm of AuNP-based nucleic acid detection methods, the principle of the sandwich hybridization strategy is reported in Figure 4.

Briefly, AuNPs are conjugated to antisense oligonucleotides (ASOs), typically thiol-modified single-strand DNA molecules with a sequence complementary to the target nucleic acid. In the simplest design, two AuNP–ASO sets are synthesized in such a way that they hybridize to two distinct and adjacent sequences on the target molecule. Whereas, in absence of the target nucleic acid, the solution containing the AuNP–ASO mixture will appear bright red due to the typical SPR band of dispersed gold colloids, in the presence of DNA complementary to the two ASOs and at a temperature T below the melting temperature Tm of both ASO target DNA duplexes, the AuNP-ASOs will aggregate and cause optical transition (Figure 4).

This approach to the detection of nucleic acids, which only relies on the simple mixing of reagents and a simple visual readout, has been adopted since the earliest AuNP-based plasmonic biosensors [18] and has been recently proposed as a clinical diagnostic tool for the point-of-care diagnosis of SARS-CoV-2 infections, highlighting the applicability to both DNA and RNA detection [9].

One of the limits of this approach is that the change in color becomes evident only if the proportion of aggregated AuNPs is sufficiently high to eliminate or at least mask the plasmonic band. At very low concentrations of the target DNA, this is unlikely the case, thus limiting the sensitivity of this method, especially when compared to the extremely low limit of detection (LOD) achievable using conventional molecular diagnostic techniques based on the enzymatic amplification of the DNA, such as the polymerase chain reaction (PCR). On the other hand, PCR is time-consuming as it requires multiple temperature cycles and can only be performed in laboratories that are equipped with a thermocycler and dedicated readout instrumentation (e.g., gel electrophoresis apparatus or fluorescence-based real-time PCR instruments).

**Figure 5:**
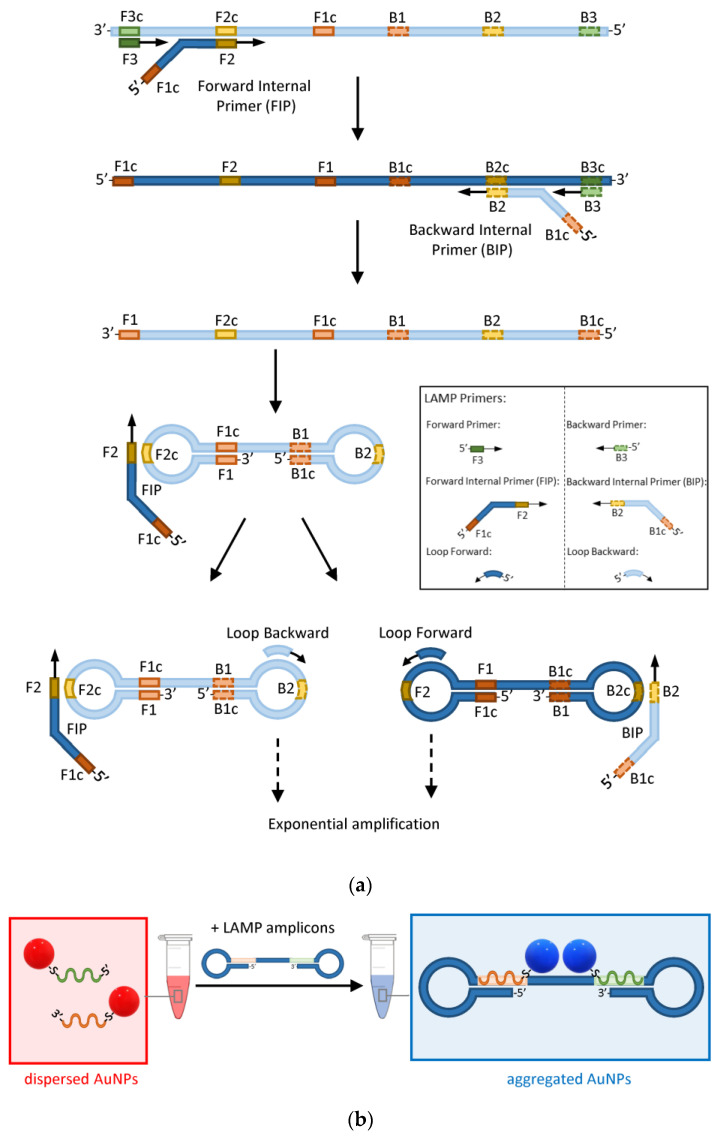
The principle of AuNP-enhanced LAMP assays. (**a**) Schematic of the main steps involved in the amplification of DNA by LAMP. Light and dark blue represent complementary strands. Green, orange, and yellow rectangles represent the forward (solid outline) and backward (dashed outline) primers’ target sequences and their complements identified by the letter ‘c’. (**b**) Detection of LAMP amplicons by hybridization of a pair of AuNP–ASOs to complementary regions on the target DNA (orange and green rectangles).

A recent implementation of the sandwich hybridization strategy combines the point-of-care capability of AuNP-based plasmonic biosensors with the extreme sensitivity of loop-mediated isothermal nucleic acid amplification (LAMP) [12]. LAMP uses a polymerase that has the ability to displace any DNA that it finds on its way while synthesising the complementary strand [62]. Therefore, using a combination of forward and backward primers and additional internal primers that can anneal at multiple locations of the target DNA, the polymerase synthesizes double-loop structures that can sustain exponential amplification without the need for alternating cycles of melting and annealing temperatures as in the conventional amplification by PCR (Figure 5a).

Opposite to PCR, LAMP happens at a constant temperature of about 65°C and therefore does not require a thermocycler. A pair of forward and backward loop primers can be also used to make the amplification faster [63]. LAMP is so efficient that it does not generally require the preliminary purification of nucleic acids. These characteristics make LAMP ideal for point-of-care testing, provided that methods to visualise the presence of LAMP amplicons by the naked eye are made available. Such methods, based on sandwich hybridization of AuNP–ASOs, have been developed and represent unique opportunities to combine the extreme sensitivity of LAMP or other isothermal amplification methods with the portability of AuNP-based biosensors (Figure 5b) [12,64,65].

Adding a retro-transcriptase to the LAMP mixture also allows for the detection of RNA targets via the generation of a cDNA template as in the case of the detection of RNA viruses such as SARS-CoV-2 for which a limit of detection of 10 copies of target RNA/µL of clinical samples was reported using AuNP–ASOs for visual readout [12].

**Figure 6:**
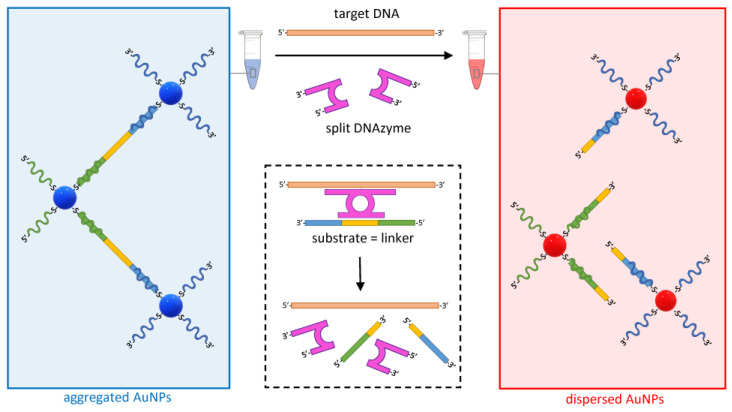
Dispersion of AuNPs mediated by the target-specific activation of a split DNAzyme (purple). The hybridization of designer arms to the target DNA (orange) triggers the assembly of the DNAzyme and the activation of the catalytic arms that selectively cleave a complementary DNA sequence within the linker (DNA substrate in yellow). The cleavage of the linker promotes the dispersion of the otherwise complexed AuNPs. As the target DNA and the split DNAzyme are available for another cleavage cycle, the dispersion of AuNPs is sustained.

Another approach to detecting specific DNA targets is based on the use of hybridization-activated DNAzymes, catalyzing the cleavage of a DNA linker that holds AuNPs together, causing their dispersion and a shift in the solution color to red (Figure 6) [54].

DNAzymes are synthetic DNA enzymes with the ability to cleave another DNA molecule. In the assay design illustrated in Figure 6, the DNAzyme is initially split into two inactive halves, each including an arm designed to be complementary to adjacent target DNA sequences. In the presence of the specific target DNA, the two halves assemble into an active form of the DNAzyme that hybridizes and cleaves its specific DNA substrate, leading to the dispersion of AuNPs. Whereas, in the LAMP-based example above, the sensitivity of the assay is given by the amplification of the target nucleic acid, here, the efficiency is driven by the fact that, as in the case of a conventional enzyme, the DNAzyme is available to catalyze new cleavages of the substrate indefinitely, causing a substantial change in the aggregation state of the colloid even at low concentrations of the target. Using this approach, a sensitivity of 50 pM was reported when detecting DNA targets from a range of bacterial, viral, and parasitic pathogens [54].

### 4.2. Detection of Proteins

The first reported AuNP-based ultrasensitive protein assay was aimed at detecting the prostate-specific antigen (PSA) [66]. It combined the recognition of the PSA by AuNP-immobilized specific antibodies with the encoding of the AuNP probes using immobilized DNA barcodes. The PSA-bound AuNPs were harvested by magnetic particles, also functionalized with anti-PSA antibodies, and all excess molecules were then washed away following the application of a magnetic field at the bottom of a test tube. In presence of PSA in the analyzed sample, the DNA barcodes on the magnetically retained AuNPs could be detected either by hybridization to a surface functionalized with DNA complementary to the barcodes (30 aM sensitivity) or by PCR (3 aM sensitivity). The use of DNA barcodes enables remarkable sensitivity while also providing potential multiplex capabilities. Following the development of this method, several other designs were reported for the detection of PSA or other proteins, some achieving a similar LOD [67].

**Figure 7:**
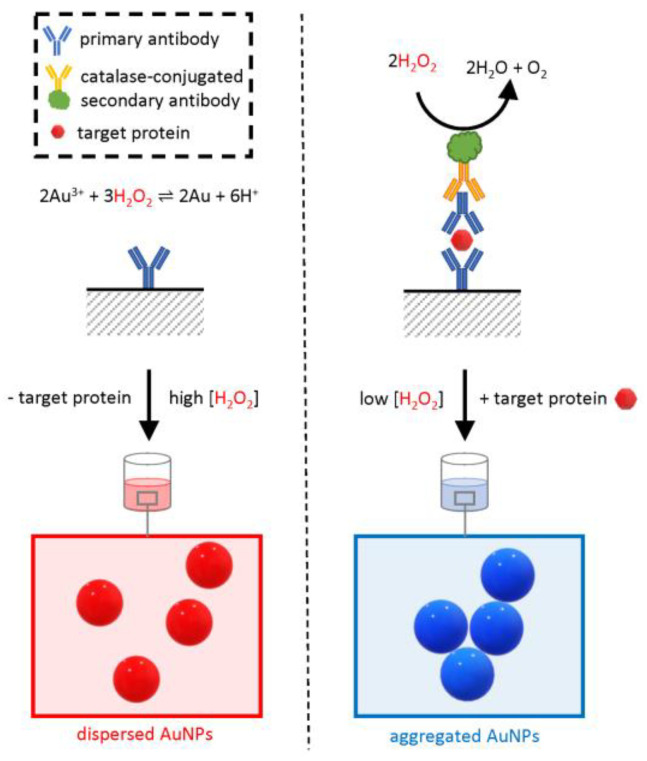
Schematic of the working principle of plasmonic ELISA. The assay is similar to a conventional double-sandwich ELISA; however, the readout is based on the in situ synthesis of AuNPs by gold (Au) reduction due to hydrogen peroxide (H_2_O_2_). The synthesized AuNPs confer a bright-red color to the test solution. Instead, when the target protein is present, the enzyme catalase is retained in the test well and catalyzes the removal of H_2_O_2_, causing the formation of an aggregated blue colloid.

Among these methods, a variant of the common enzyme-linked immunosorbent assay (ELISA) used an AuNP-based readout in place of the conventional colorimetric substrates and was therefore named “plasmonic ELISA” (Figure 7) [55,68].

Similar to conventional ELISA, the assay relies on a specific antibody adsorbed on a surface, typically the bottom of a plastic multi-well plate. If present, the analyte binds in a “sandwich” configuration to the immobilized antibody and an extra primary antibody in the test solution. The test solution also includes an enzyme-conjugated secondary antibody that binds to the primary antibody. Following a stringent washing step, the enzyme is retained on the surface only if the target protein was originally present in the sample, and the amount of enzyme in the well is proportional to the original analyte concentration. The result of the catalytic activity of this enzyme provides an amplified readout of the presence of the target protein.

Whereas, in a conventional ELISA, the enzyme would be a peroxidase or a phosphatase with the ability to change the absorbance of a colorimetric substrate read by a spectrophotometer, in plasmonic ELISA, the naked-eye readout is based on a change in colloidal dispersion of AuNPs mediated by the enzymatic activity of catalase. Opposite to the plasmonic assays described so far, here there is no addition of premade functionalized AuNPs, but instead, they are synthesized by a hydrogen peroxide-mediated reduction of gold ions added to the test solution. When the concentration of hydrogen peroxide is sufficiently high, the synthesis of AuNPs results in a well-dispersed, homogenous, intensely red colloid with a clear plasmonic band. However, when in positive samples, the concentration of hydrogen peroxide is lower due to the catalytic activity of catalase, the reduction of gold ions is less efficient, and the resulting colloid is irregular, lacking a clear plasmonic band and with a blue-shifted overall color. The catalase-mediated effect on the colloidal structure is proportional to the concentration of the analyte in the original sample allowing for the quantitative determination of the analyte concentration if a standard curve is available. The method was first applied to detect PSA with an LOD of 1 × 10^−18^ g/mL [55].

It is important to note that this method does not require complex bioconjugation of AuNPs to biomolecules, such as DNA oligonucleotides or antibodies, although bespoke cross-linking of catalase to the secondary antibody is required and therefore only relies on readily available reagents, making it particularly accessible to laboratories that already have the expertise to perform conventional ELISA [68].

**Figure 8:**
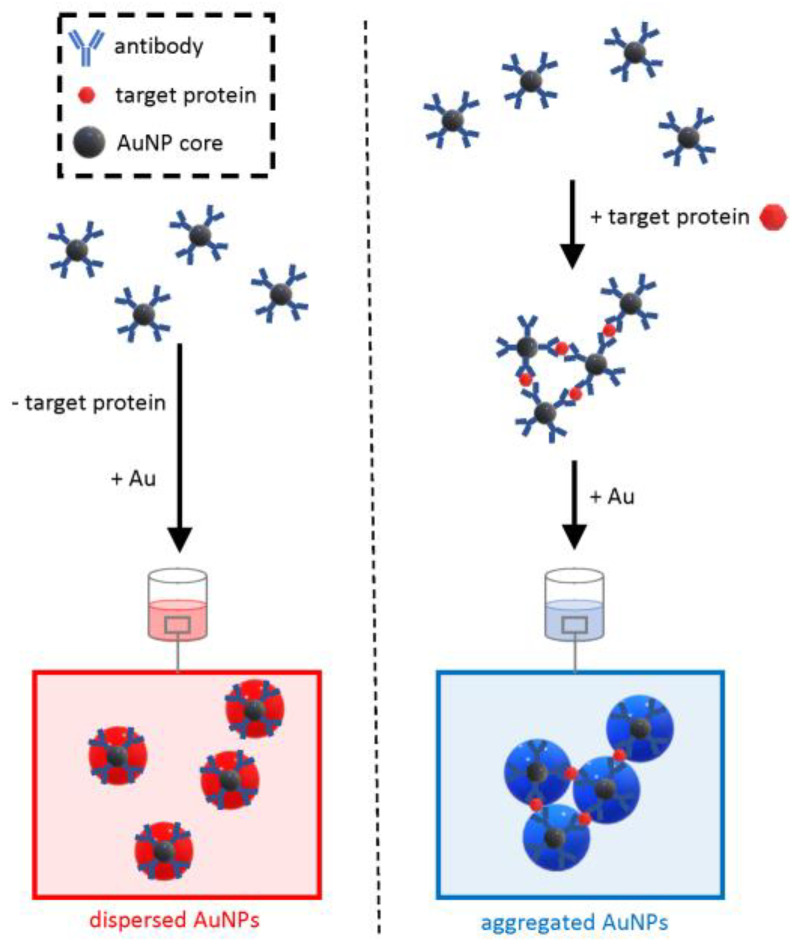
Schematic of the working principle of the wash-free plasmonic immunoassay. The method relies on the growth of antibody-conjugated core AuNPs as a dispersed colloidal suspension (negative sample) or aggregated clusters (positive samples).

On the other hand, due to the number of steps required, including antibody adsorption on plates and several washing steps, the technique cannot be entirely considered an “in solution” assay that only requires mixing followed by rapid readout. To address this limitation, a wash-free plasmonic immunoassay was developed that was able to detect various cancer biomarkers within 30 s with an LOD of less than 2 × 10^−9^ g/mL (Figure 8) [56].

This approach relies on the formation of cross-linked clusters of antibody-functionalized AuNPs when the target protein is “sandwiched” in between antibodies. The functionalized AuNPs have a core of moderate diameter (15 nm), and at low concentrations of the analyte, it would be hard to distinguish by the naked eye the optical transition from the red color of a dispersed colloid to the blue of aggregated clusters. To increase the sensitivity, the detection method exploits extra growth of the core AuNPs at the expense of gold ions dissolved in the solution. The modulation of the interparticle spacing of AuNP oligomers controlled by the growth of the gold allows for a quick, visible readout in the form of a sharp optical transition from red to blue in the presence of the target protein. Opposite to the plasmonic ELISA, this approach does not need laborious washing steps but, on the other hand, requires the bespoke synthesis of antibody-conjugated core AuNPs. The simple and rapid testing procedure makes this approach an ideal candidate for deployable test kits.

### 4.3. Detection of Other Analytes

Some of the designs described above have been also used for the detection of small molecules or ions. The DNAzyme approach depicted in Figure 6 has been used for detecting lead ions (Pb^2+^) with an LOD of 100 nM [57]. In this biosensor, the DNAzyme is not split and is designed to hybridize to its substrate, which is within the linker that holds the AuNP clusters together. The DNAzyme is activated by Pb^2+^ and cleaves the linker, causing the dispersion of the AuNPs and an optical transition of the solution towards red.

Mercury ions (Hg^2+^) were detected with an LOD of 100 nM using an approach similar to the one depicted in Figure 4 [58]. Instead of allowing sandwich hybridization on a target DNA, the two oligos immobilized on AuNPs were designed to be complementary to each other, except for a thymidine-thymidine (T-T) mismatch. In a negative sample, the mismatch would be sufficient to prevent hybridization and would yield a dispersed colloid with a bright-red color. However, due to the specific coordination of Hg^2+^ between the two thymidine molecules (T-Hg^2+^-T), in presence of Hg^2+^, the colloid aggregates and the solution become blue.

**Figure 9:**
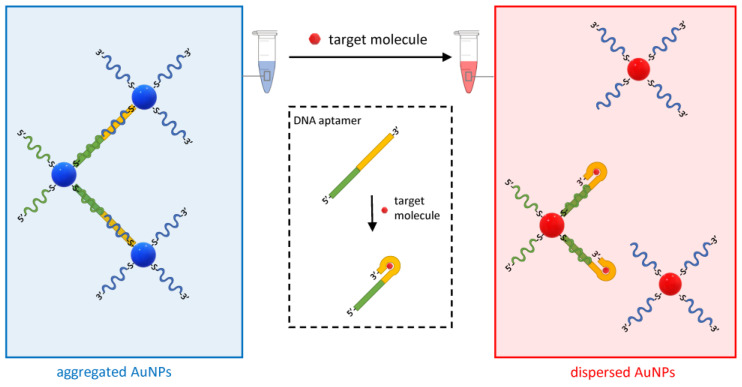
Schematic of the working principle of the aptamer-based plasmonic assay. The method uses DNA aptamers hybridized to DNA oligos on two sets of AuNPs. The aptamer undergoes a conformational change when bound to the analyte, which causes the de-hybridization of one of the complementary DNA oligos and the subsequent dispersion of the AuNPs.

Another variant of the approach in Figure 6 based on DNA aptamers has been developed for various analytes (Figure 9) [69]. DNA aptamers are single-stranded oligonucleotides that bind target molecules with high affinity and specificity. They are obtained by the systematic evolution of ligands by exponential enrichment (SELEX), which is an efficient combinatorial method that starts from random DNA sequences that “evolve” over successive steps to bind increasingly better to the target molecule [70].

In the assay in Figure 9, two DNA oligo-modified AuNP sets are designed to hybridize to the DNA aptamer and form AuNP clusters. However, when the target molecule is present in the solution, its binding to the DNA aptamers displaces one of the hybridized oligos and disperses the clusters. This method was initially developed to detect adenosine and cocaine with an LOD of 300 and 50 µM, respectively, but was then expanded to any analyte for which it is possible to obtain a functional aptamer [57,59].

## 5. Discussion, Conclusions, and Future Directions

This review describes a selection of methods for the rapid testing of biomolecules and other analytes using a combination of innovative molecular mechanisms and visual readout based on the unique optical properties of AuNPs. It is beyond the scope of this review to give a comprehensive overview of all plasmonic biosensors as it is focused on those that make use of spherical AuNPs and only rely on the simple mixing of solutions. For example, plasmonic biosensors that use alloys of gold with other noble metals, nanomaterials with non-spherical shapes such as nanorods, electrochemical readout, gold layers, gold-functionalized fiber probes, surface-enhanced Raman scattering, microfluidic, lateral flow, and lab-on-a-chip devices are not covered here [71,72,73,74,75,76]. The selected examples described also report the sensitivity achieved by each method, which highlights the difficulty to compare the LOD across different techniques due to the use of different targets and different units [67].

The lack of benchmarks and unified reporting of performance may indicate that, despite two decades of research, the field of AuNP-based rapid assays is still young. Another indication that the application of plasmonic biosensors is still in its infancy is the fact that the approaches described in this review represent pilot research studies with no substantial evidence of commercialization and reproducibility [77]. In fact, current commercial rapid tests for molecular diagnostics are predominantly based on well-established LFAs. However, the confidence in LFAs built during the COVID-19 pandemic is likely to fuel an increase in the demand for self-tests in healthcare, paving the way to a potential differentiation in the technologies in use. Future commercial products based on innovative biosensor approaches will also need to take into account the environmental impact of the large-scale use of disposable devices, possibly favoring methods which make use of eco-friendly materials [78].

The demand for deployable tests beyond clinical diagnostics is emerging, as evidenced by research in areas such as water quality, aquaculture, and environmental monitoring [79,80,81], food and beverage quality and authentication [82,83,84], detection of food allergens [85], and detection of pyrogens such as endotoxin in biotechnology products [86]. In conclusion, as a paradigm of the inventive use of plasmonic biosensors, a recent niche application is worth mentioning: the rapid determination of whisky age by SPR [87]. It was found that whisky itself promotes the formation of plasmonic AuNPs by the reduction of gold ions mixed in a solution containing whisky. The aggregation state and the shape of the SPR peak broaden as a result of maturation in casks, likely due to the presence of gallic and tannic acids, which affect the kinetics of the formation of colloidal gold. This example highlights the potential of rapid plasmonic assays for applications in many areas yet to be explored.

## Figures and Tables

**Table 1:** Optical properties of the AuNP colloids from Figure 2. d, λ_max_, ε, and [AuNP] represent the diameter, extinction peak, extinction coefficient at the peak, and concentration required to achieve an extinction of 1 OD over a 1 cm path length, respectively. The values are calculated from the information on the product datasheets (nanoComposix, San Diego, CA, USA).

d (nm)	λ_max_ (nm)	ε (M^−1^ cm^−1^)	[AuNP]_OD=1_
5	515	7.40 × 10^6^	135 nM
10	517	1.32 × 10^8^	7.55 nM
15	518	3.73 × 10^8^	2.68 nM
20	520	9.17 × 10^8^	1.09 nM
30	520	3.33 × 10^9^	300 pM
40	523	8.86 × 10^9^	113 pM
50	527	1.94 × 10^10^	51.4 pM
60	533	4.29 × 10^10^	23.3 pM
70	539	5.60 × 10^10^	17.9 pM
80	544	7.83 × 10^10^	12.8 pM
100	562	1.25 × 10^11^	8.00 pM

**Table 2:** Summary of selected representative methods of detection detailed in the following sections. The limit of detection (LOD) is defined as the lowest assayed concentration of analyte that yields a signal higher than three times the standard deviation of the negative controls expressed in the units used in the relevant references.

Molecule/s	Representative Detection Method	Reported LOD	Target	Reference
Nucleic acids	Sandwich hybridization	10 copies of target RNA/µL	SARS-CoV-2	[12]
Nucleic acids	DNAzyme hybridization	50 pM	DNA from various pathogens	[54]
Proteins	Plasmonic ELISA	1 × 10^−18^ g/mL	Prostate-specific antigen (PSA)	[55]
Proteins	Wash-free plasmonic immunoassay	2 × 10^−9^ g/mL	Various cancer biomarkers	[56]
Ions	DNAzyme hybridization	100 nM	Lead ions (Pb^2+^)	[57]
Ions	Sandwich hybridization	100 nM	Mercury ions (Hg^2+^)	[58]
Organic molecules	Aptamer-based plasmonic assay	300 µM	Adenosine	[59]
Organic molecules	Aptamer-based plasmonic assay	50 µM	Cocaine	[59]

## Data Availability

No new data were created or analyzed in this study. Data sharing is not applicable to this article.
